# Small airway dysfunction and bronchial asthma control : the state of the art

**DOI:** 10.1186/s40733-015-0013-3

**Published:** 2015-12-01

**Authors:** Marcello Cottini, Carlo Lombardi, Claudio Micheletto

**Affiliations:** 1 Allergy and Pneumology Outpatient Clinic, Bergamo, Italy; 2 Departmental Unit of Allergology, Immunology & Pulmonary Diseases, Fondazione Poliambulanza, Via Bissolati, 57, Brescia, 25124 Italy; 3 Respiratory Unit, Mater Salutis Hospital, Legnago, Verona Italy

**Keywords:** Small-airways disease, Bronchial asthma, Phenotypes, Asthma control

## Abstract

According to national and international guidelines, achieving and maintaining asthma control is a major goal of disease management. In closely controlled clinical trials, good asthma control can be achieved , with the medical treatments currently available, in the majority of patients , but large population-based studies suggest that a significant proportion of patients in real-life setting experience suboptimal levels of asthma control and report lifestyle limitations with a considerable burden on quality of life. Poor treatment adherence and persistence, failure to use inhalers correctly, heterogeneity of asthma phenotypes and associated co-morbidities are the main contributing factors to poor disease control. Now, it is widely accepted that peripheral airway dysfunction , already present in patients with mild asthma, is a key contributor of worse control. The aim of this paper is to investigate the association between small-airways dysfunction and asthma symptoms/control. We therefore performed a PubMed search using keywords : small airways; asthma (limits applied: Humans, English language) and selected papers with a study population of asthmatic patients, reporting measurement of small-airways parameters and clinical symptoms/control.

## Background

Asthma is one of the most common chronic conditions in the world and the most common non-communicable disease among children [1]; according to the World Health Organization , the Global Burden of Disease Study and the Global Asthma Report 2014 [2–4], asthma affects an estimated 334 million people worldwide. The prevalence of asthma has been reported to range from 1 to 18 % of the population in different countries [4]. Most people affected are in low- and middle-income countries, and the prevalence of asthma is estimated to be increasing fastest in those countries [4]. In Europe alone, asthma affects 30 million people [5, 6] and is associated with a significant socioeconomic burden [7, 8]. The Global Burden of Disease Study estimated that asthma was the 14th most important disorder in terms of global years lived with disability [3].

The main goal of current asthma treatment guidelines is to achieve clinical control, including control of symptoms (daytime symptoms, night-time awakenings and reliever inhaler use) , maintenance of normal activity levels and to prevent exacerbations [9, 10].

Randomized controlled trials showed that asthma control is an achievable target [11] , but the incidence of asthma control in “real-life” clinical practice is considerably lower and a substantial proportion of asthmatics remain not well-controlled [12–14]. Randomized controlled trials (RCT) are not representative of real-life, because recruitment often includes only patients with no (or negligible) co-morbid illnesses or concurrent medications, those with good inhaler technique and high adherence to study therapies [15]. Lifestyle characteristics, as cigarette smoking, typically result in patient exclusion. The level of asthma control is poor even in patients with mild asthma, regularly treated with inhaled steroids [16]. Poor asthma control is associated with increased risk of exacerbations, impaired quality of life, increased health-care utilization and reduced productivity [17–19]. History of asthma exacerbations, poor treatment adherence, failure to use inhalers correctly, heterogeneity of asthma phenotypes and associated comorbidities are the main contributing factors to poor disease control [20–24]. Recent studies suggest that persistent uncontrolled inflammation in the peripheral small airways can also contribute to clinical expression and worse control of asthma [25]. Historically, the small airways are defined as airways with an internal diameter of less than 2 mm that do not contain cartilage in their walls and extend from the 8th generation airways to the periphery of the lung, referring to the landmark study of Macklem and Mead [26]. It is well established that inflammation and remodeling in asthma involve the large airways, but it is now widely accepted that small airways are the major site of inflammation in asthma [27], with a chronic inflammatory infiltrate consisting of eosinophils, T lymphocytes, neutrophils, and macrophages; moreover trans-bronchial biopsy findings show small airways inflammation and remodeling in all severities of asthma [27–30]. The small airways are known as the “quiet zone” because they make only a small contribution to airway resistance under normal circumstances. Conventional physiological measurements are unable to sensitively evaluate this airway region [31, 32] and may become abnormal only once there is a significant burden of disease, but in recent years more specialized tests have been developed, which may better assess small-airways dysfunction. These tests are now moving from clinical research laboratories into routine clinical practice. Table 1 summarizes the techniques available for the assessment of small airways disease. No assessment method is universally and directly representative of peripheral airway function and the value and limitations of each test have been extensively reviewed [33–35].

**Table 1:** Techniques available for the assessment of small airways disease in comparison to large airway

Method	Small airway function	Large airway function
Spirometry	FEF25–75 %, FVC, FVC/SVC	FEV1, FEV1/FVC
Impulse oscillometry (IOS)	R5–R20, X5, AX, Fres	R20
Single Breath Nitrogen Washout (SBNW) or Multiple Breath Nitrogen Washout (MBNW) test	Slope phase III, CV, CC, Sacin, Scond	
Body plethysmography	RV, RV/TLC	
High Resolution Computerized Tomography (HRCT)Nuclear medicine (Scintigrapy,SPECT,PET)3He-MRI	Air trapping, airway wall thicknessRegional ventilation defectsNonventilated lung volume	Airway wall thickness
Bronchoscopy	Transbronchial biopsy, BAL	Endobronchial biopsy
Sputum induction	Late phase sputum	Early phase sputum
Exhaled nitrix oxide (eNO)	Alveolar eNO	Bronchial eNO
CT & computational fluid dynamics	Changes in airway volume and resistence	

Evidence is accumulating to support a high prevalence of impaired small airway function in patients with asthma. Anderson and Colleagues [36] studied with impulse oscillometry (IOS) the prevalence of small airways dysfunction (SAD) in 368 patients with community managed persistent asthma who were receiving treatment as defined by British Thoracic Society (BTS). An abnormal value for peripheral airways resistance (defined as R5–R20 higher than 0.03 kPa/ l^−1^) was noted in 65 % of patients on step two BTS treatment, 64 % of patients on step three treatment, and 70 % of patients on BTS step four treatment. Perez and colleagues [37] studied 441 patients with moderate-to-severe asthma with normal FEV1 and FEV1/FVC, treated with an association of ICSs and LABAs . The prevalence of SAD was estimated by both spirometry and plethysmography and defined by the presence of the following parameters: (1) the difference between SVC and FVC to detect expiratory air trapping; (2) FEF _**25–75 %,**_ and FEF _**50 %,**_ to detect distal airflow limitation; (3) functional residual capacity, RV, and RV/TLC as marker of air trapping/lung hyperinflation, a phenomenon closely associated with small airways dysfunction (premature small airways closure or near closure: the small airways begin to collapse at a higher volume before expiration is complete). SAD was found in more than half of the patients indicating that the routinely used lung function tests can underestimate dysfunctions occurring in the small airways. Recent studies suggest that abnormalities in the small airways can contribute to the clinical expression of asthma [34, 35, 38] and a systematic review showed that SAD is associated with worse asthma control, a higher number of exacerbations, the presence of nocturnal asthma, more severe bronchial hyperresponsiveness (BHR) and exercise-induced asthma [25]. Air trapping/lung hyperinflation are characteristic features of the severe asthma population [29, 85, 88] indicating an additional potential severe asthma phenotype [29].

### Small airways dysfunction and asthma symptoms

Several studies have linked small airway function to asthma symptoms. Recently, Schiphof-Godart et al. selected patient with SAD, based on FEF _**50 %,**_ and R5–R20 values from spirometry and IOS respectively [39]. Patients with SAD reported to wheeze easily, were unable to breathe in deeply, mentioned more symptoms related to BHR, experienced more pronounced exercise-induced symptoms and more frequently had allergic respiratory symptoms after exposure to allergens. According to asthma treatment guidelines [9, 10], the presence of night-time awakenings and activity limitation due to exercise-related symptoms is a key factor for worse asthma control. A history of EIB and exercise-related respiratory symptoms occur more commonly in patients with not well and very poorly controlled asthma. Small airways involvement has been implicated in exercise-induced asthma (EIA) and in the severity of exercise-induced bronchoconstriction (EIB). Kaminsky et al. reported that the small airways of patients with EIA are responsive to cool, dry air, which suggests that they play an important role in asthma in this patient population [40]. The Authors also reported that baseline peripheral airway resistance correlated significantly with the degree of EIB in asthmatic patients. Decramer et al. showed that peripheral resistance, as measured with the forced oscillation technique, increases after a hyperventilation test with cold, dry air [41]. This alteration is compatible with a more generalized constriction of the peripheral as well as central airways. In asthmatic children, FEF _25–75 %,_ a parameter of small airways function, decrease in response to exercise without changes in FEV1, mainly in patients with mild asthma [42]. Testing mild asthmatic patients for airway hyperresponsiveness with indirect stimuli, Aronsson et al. [43] showed that those positive to mannitol had a significant increased baseline value of IOS parameters such as ΔR5-R20 and reactance area (AX). Lee et al. evaluate the characteristics of airway obstruction in young asthmatics after an exercise bronchial provocation test using IOS : more severe exercise-induced bronchoconstriction is associated with a higher increase in peripheral airways resistance (R5–R20) but not with an increase in large-airways resistance (R20) [44]. Two studies investigated the phase III slope of the single-breath washout test before and after a cold, dry air hyperventilation test and demonstrated that an increase in the helium and sulfur hexafluoride phase III slopes were associated with the degree of EIB in the asthma patients [45, 46]. Chinellato et al. assessed the correlation between alveolar production (C_**alv**_NO) and bronchial flux (J(NO) of nitric oxide and EIB in asthmatic children [47]. A significant correlation was observed between severity of EIB and inflammation of the central and peripheral airways. Linkosalo et al. showed that in atopic children and adolescents increased alveolar NO concentration correlated with the degree of obstruction in smaller airways induced by exercise challenge [48]. Taken together, these findings support the view that inflammation and dysfunction in the peripheral airways are crucial for more pronounced exercise-induced symptoms in asthma.

Nocturnal symptoms and worsening of lung function at night are common among patients with asthma and are associated with poor asthma control. Several studies investigate the association between SAD and nocturnal asthma. Kraft and Colleagues have reported that patients with nocturnal asthma demonstrate increase in peripheral airways resistance and greater inflammatory involvement of the small airways [49, 50]. Patients with nocturnal asthma exhibited significantly greater numbers of eosinophils in the distal airways compared to the proximal airway tree in biopsies undertaken during the night and in bronchoalveolar lavage fluid [50, 51]. Lehtimaki et al. evaluate patients with newly-diagnosed steroid-naive asthma, assessing alveolar NO concentration and bronchial NO flux in 40 asthmatics and 40 healthy controls [52]. Patients with nocturnal symptoms had a higher alveolar NO concentration (1.7 ± 0.3 parts per billion (ppb)) than patients without nocturnal symptoms (0.8 ± 0.3 ppb, *p* = 0.012) or healthy controls (1.0 ± 0.1 ppb, *p* = 0.032), suggesting that even in patients with mild asthma, nocturnal symptoms are associated with small-airways inflammation. Taken together, these studies support the concept that SAD may contribute to the increased night-time symptoms in patients with nocturnal asthma.

Obesity has been linked with asthma symptoms, need for asthma treatment and reduced lung function [53]. Recently, Al-Alwan et al. evaluated lung function by conventional clinical tests and by impulse oscillometry in female late-onset, non-allergic patients with asthma and control subjects before, and 12 months after, bariatric surgery [54]. Weight loss decompresses the lung in both obese control subjects and patients with asthma, but there was a significantly different response to weight loss in patients with asthma compared with control subjects, and this result lead the Authors to the novel hypothesis that obese patients with asthma are distinguished from obese control subjects by having excessive collapsibility of the lung periphery, perhaps as a consequence of reduced distal airway wall stiffness.

### Small airways dysfunction and asthma control

Takeda et al. performed IOS, spirometry, assessment of health status (Asthma Quality of Life Questionnaire and St. George’s Respiratory Questionnaire), dyspnea (Baseline Dyspnea Index) and disease control (Asthma Control Questionnaire) in 65 patients with stable asthma [55]. Small airway function as evaluated by peripheral airway IOS indices, correlated better with clinical symptoms and asthma control than spirometry; furthermore, greater small-airways reactance was associated with loss of asthma control. Pisi et al. investigated the presence of SAD based on increased peripheral airway IOS indices in 33 adult asthmatic patients with normal FEV1 values [56]. Small airway dysfunction, as assessed by IOS and spirometry, was associated with poor disease control, assessed by the Asthma Control Test. Recently, Manoharan and Colleagues valuated adult asthmatics with a preserved FEV1 (>80 % predicted) [57]. Spirometry and IOS measurements were linked to prescription data. Persistent small airway dysfunction, defined by FEF _**25–75 %,**_ and R5-R20, was associated with a significantly increased likelihood of having worse long-term asthma control. The risk of having poorer control was greater when measurements of FEF _**25–75 %,**_ and R5-R20 were combined. These studies support the concept that in adult asthmatics who have a preserved FEV1, a persistent small airway dysfunction is associated with poorer control, perhaps suggesting the presence of a defined “***small airway asthma phenotype”*** characterized by individuals with healthy values for conventional measures of pulmonary function but poor control of disease and a disproportionate amount of small airway dysfunction [58]. This situation is very common in childhood, where FEV1 is generally normal, even in severe persistent asthma [59]. Several studies in children have linked uncontrolled asthma to small airway function. Rao et al. used The Children’s Hospital Boston Pulmonary Function Test database to compare matched groups of children with asthma [60]. Subjects with a low FEF _**25–75 %,**_ had a worse control of asthma and more exacerbations . Shi et al. assessed 57 children with controlled asthma and 44 children with uncontrolled asthma with spirometry and IOS: they found that small airway IOS measurements (R5–R20 and reactance area values) could discriminate between patients with controlled and uncontrolled asthma, with a high sensitivity and specificity of 84 and 86 % [61]. The sensitivities of spirometry outcomes for assessing uncontrolled asthma were low, especially for FEV1 and bronchodilator responsiveness. In a prospective follow-up study of the same group [62], children with controlled asthma who have increased peripheral airway IOS indices (reactance area >0 · 70 kPa/L and R5–R20 > 0 · 10 kPa/ l^−1^) are at risk of losing asthma control.

Many studies assessed ventilation heterogeneity: increasing unevenness of ventilation between different lung regions is a sensitive marker of abnormal small-airway function and can be measured noninvasively by using the single-breath washout (increase in the phase III slope, dN2) or multiple-breath washout techniques (MBNW) [61–66]. MBNW is able to distinguish between ventilation heterogeneity generated in the conductive lung zone (Scond) and ventilation heterogeneity generated in the acinar lung zone (Sacin) [32]. Bourdin et al. demonstrated that patients with more alveolar heterogeneity, as determined with the phase III slope of the single-breath nitrogen test (SBNT), have worse asthma control [67]. Singer et al. assessed ventilation heterogeneity with an easy tidal breath single-breath washout (SBW) technique in school-aged children with mild asthma and normal FEV1 and healthy age-matched control subjects [68]. Abnormal acinar ventilation heterogeneity in one-third of the children suggests that small airways disease may be present despite mild asthma symptoms and normal spirometry. Farah et al. demonstrated in a cross-sectional analysis of a large cohort that subjects with poorly controlled asthma had worse ventilation heterogeneity compared with well-controlled subjects [69]. Furthermore, during a period of ICS treatment, the change in ventilation heterogeneity predicted the change in asthma symptom control independently of all other measured physiologic variables. The same group demonstrated that ventilation heterogeneity predicts symptomatic improvement to ICS dose up-titration and loss of symptom control during down-titration [70]. Recently Thompson and Colleagues, using the multiple-breath washout techniques (MBNW), demonstrated that in patients with poorly controlled asthma a functional abnormality in the acinar lung zone showed a direct correlation with airflow obstruction and treatment requirement [71].

With regard to peripheral inflammation, Van Vyve et al. investigated inflammation in bronchoalveolar lavage (BAL) fluid [72]. Uncontrolled asthma was associated with a higher eosinophil percentage in BAL fluid, suggesting involvement of the small airways.

Several studies in children and adults have demonstrated that higher alveolar nitric oxide concentrations are associated with the presence of worse asthma control [72–76]. Corcuera-Elosegui et al. recently assessed 162 children with spirometry, exhaled NO at multiple flow and asthma control questionnaire (ACQ): FEV1/FVC decreased significantly and morbidity was significantly higher in asthmatics with elevated alveolar nitric oxide concentrations [77]. Puckett et al. measured baseline spirometry, bronchodilator response, eNO at multiple flows (50, 100, and 200 ml/s) , asthma control and morbidity in 200 children with asthma and 21 non-asthmatic, non-atopic controls and divided children into 4 groups based on the concentration of alveolar and bronchial NO: only categories with increase alveolar nitric oxide concentrations were related to poor asthma control and morbidity independent of baseline spirometry, bronchodilator response, atopic status, or use of inhaled corticosteroids. Scichilone and Colleagues utilized the index of alveolar nitric oxide as a marker of small airways inflammation in patients with mild asthma and established that the level of disease control, assessed using the asthma control test (ACT), was directly associated with peripheral airways inflammation: in uncontrolled asthmatic patients compared to controlled patients, worsening alveolar nitric oxide concentrations correlated with worsening ACT scores [79].

### Small airway disfunction and asthma exacerbations

The focus of clinical practice guidelines for asthma [9, 10] has shifted to include not only the conventional assessment of symptoms, reliever use, and activity limitation but also assessment of the patient’s future risk of adverse outcomes, such as exacerbations, future poor asthma control, accelerated decline in lung function, and adverse effects of medications. Uncontrolled asthma symptoms substantially increase the risk of exacerbations [80, 81], but data from the European Network for Understanding Mechanisms of Severe Asthma (ENFUMOSA) found that patients with a history of near-fatal asthma in the past 5 years could not be reliably distinguished from those with mild to moderate asthma in stable conditions using common measures of asthma severity and control [82]. Identification of patient’s risk profile is important to enable recognition of patients at high risk. Risk factors for exacerbations include: history of severe exacerbation, uncontrolled asthma symptoms, having ≥1 exacerbation in last 12 months, low FEV1, incorrect inhaler technique and/or poor adherence, smoking, obesity, pregnancy and blood eosinophilia [83].

Accumulating evidence suggests that a higher degree of peripheral airway dysfunction is associated with more frequent asthma exacerbations. Rao et al. showed that asthmatic children with a low FEF _**25–75 %,**_ had nearly 3 times the odds (OR 2.8, *p* < 0.01) of systemic corticosteroid use and 6 times the odds of asthma exacerbations (OR 6.3, *p* > 0.01) compared with those who had normal spirometry [60]. The Authors conclude that a low FEF _**25–75 %,**_ in the setting of a normal FEV1 is associated with increased asthma severity, systemic steroid use and asthma exacerbations in children. Pisi et al. investigated the presence of SAD by IOS in asthmatic patients with normal FEV1 values [54]. Increased R5–R20 values were significantly higher in patients with asthma exacerbations, when compared with patients without asthma exacerbations. Two studies investigated the relationship between ventilation heterogeneity and asthma exacerbations; Bourdin et al. [67] showed that frequent (>2/y) exacerbators have a higher degree of ventilation inequalities (a sensitive marker of abnormal small-airway function), as determined with SBNT phase III slope, than infrequent exacerbators (<2/y), whereas FEV1 percent predicted values were comparable between these two groups. Veen and Colleagues observed that difficult-to-treat asthmatics with frequent disease exacerbations exhibited enhanced airway closure (assessed as closing volume and closing capacity) compared to equally severe asthmatics without recurrent exacerbations [84].

In asthmatic children, Mahut et al. recorded forced expiratory flows and plethysmographic lung volumes (TLC, FRC, RV) before and after bronchodilation : air trapping (higher RV and RV/TLC) was associated with occurrence of a severe exacerbation during the last 3 months, suggesting a small airway disease that is not evidenced by forced expiratory flows [85]. Imaging with CT also allows assessment of small airways in obstructive pulmonary diseases [86]. High-resolution CT allows direct assessment of large and medium airways (diameter >2–2.5 mm), and indirect assessment of small airways. Areas of mosaic lung attenuation on inspiratory CT and air trapping on expiratory CT have been evaluated as markers of small airways disease in both asthma and COPD [87]. Busacker et al. assess with CT scanning a subset of Severe Asthma Research Program subjects; asthmatic patients with air trapping were significantly more likely to have a history of asthma-related hospitalizations, ICU visits, and/or mechanical ventilation [88]. Duration of asthma, history of pneumonia, high levels of airway neutrophils, airflow obstruction (FEV(1)/FVC) and atopy were identified as independent risk factors associated with the air-trapping phenotype. Furthermore, two studies in adults and children showed that patients with increased alveolar NO levels more frequently had visits to the emergency department, severe attacks, and hospitalizations [77, 78].

As a predictor of future risk, increased BHR appears to be, in children and adults with asthma, a significant and independent risk factor for loss of control, asthma exacerbations and development of irreversible loss of lung function [89]. Several studies have demonstrated a strong correlation between small airways dysfunction and BHR. In a landmark study, Wagner et al., using a fiberoptic bronchoscope wedged into a subsegmental bronchus, demonstrated that greater peripheral airways resistance was associated with more BHR to methacholine [90]. BHR can be present in subjects without any respiratory symptoms. In 185 subjects, Segal et al. showed that distal airway heterogeneity, as reflected by higher R5–R20 and lower X5 values, was associated with methacholine-induced symptoms despite absence of change in FEV1 [91]. Boudewijn et al. investigated small airway function assessed by spirometry and impulse oscillometry, as well as Borg dyspnea scores at baseline and during a methacholine provocation test in 15 subjects with asymptomatic BHR, 15 asthma patients, and 15 healthy controls [92]. Small airway function (R5–R20 and X5 ) was comparable between subjects with asymptomatic BHR and healthy controls, whereas asthma patients showed small airway dysfunction as reflected by higher R5–R20 and lower X5 values. Beretta et al. used the IOS to gain information concerning the distribution of hyper-reactivity along the bronchial tree during methacholine challenge test [93]. For PD20 < 200 μg, a remarkable frequency dependence was observed, with increase in R5, no change in R20, and decrease in X5, suggesting hyper-responsiveness of the distal airways paralleled by a change in visco-elastic properties of lung parenchyma. Several studies have suggested that small-airways dysfunction is associated with more severe BHR. Telenga et al. analyzed data from patients with mild-to-moderate asthma: all patients were hyperresponsive to histamine (PD20 < 9 mcg) [94]. Small airways obstruction was defined as a MEF _**50**_ of less than or the same as the lower limit of normal.The Authors found that small airways obstruction is associated with the severity of BHR in asthma, independently of FEV1. Downie et al. analyzed, in 40 subjects with asthma, airway inflammation by exhaled nitric oxide, ventilation heterogeneity by multiple breath nitrogen washout and BHR by methacholine challenge: baseline ventilation heterogeneity was a strong predictor of BHR, independent of airway inflammation in subjects with asthma [95]. While a PD20 value is measurable in the vast majority of asthma patients during the methacholine challenge, a significant ΔFVC% value is not always detectable in asthmatic patients. A fall in FVC suggests small airway closure and gas trapping: excessive bronchoconstriction during BHR testing is considered to be a very important pathophysiological determinant in severe acute asthma exacerbations [96, 97]. Furthermore, ΔFVC% correlated to asthma treatment in adult patients and to the presence of symptoms in children with asthma [98, 99]. Recently, Alfieri et al. provide the first evidence that in asthmatic patients excessive bronchoconstriction expressed by ΔFVC% is strictly associated to small airway dysfunction, as assessed by IOS [100]. As compared to patients with R5–R20 ≤ 0.030 kPa s l^−1^, patients with R5–R20 > 0.030 kPa s l^−1^ had a high likelihood to be associated to a ΔFVC% greater than 14.5 % during a methacholine-induced bronchoconstriction.

### Effect of asthma treatment on small-airways function and control of disease

Taken together, these findings support the view that distal lung is a very important target in any therapeutic strategy for effective treatment. The inability to reach and treat the peripheral airways may contribute to the lack of efficacy of inhaled treatments. Several studies have assessed the ability of both inhaled small particle aerosols and oral treatments to target the distal airways and improve physiological indices and levels of asthma control. Anti-inflammatory treatment with inhaled corticosteroids(iCSs), with or without long-acting β2-adrenoceptor agonists (LABA), is the cornerstone of asthma management [9, 10]. The recent development of inhaled extrafine formulations allows a more uniform distribution of the inhaled treatment throughout the respiratory tree to include the peripheral airways [101]. Extrafine formulations, with an mass median aerodynamic diameter (MMAD) of approximately 1 to 1.5 μm , have a higher lung deposition (50 to 60 %) than coarse particle ICSs with an MMAD of 3 to 4 μm (10 to 20 %) and then penetrate more deeply into the peripheral airways than drugs delivered via traditional inhalers [102–104].

Different biomarkers of peripheral airways are improved by extrafine inhaled corticosteroids (beclomethasone, ciclesonide, flunisolide) in comparison with equivalent non-extrafine inhaled formulations: ventilation heterogeneity [105], peripheral airways resistance [36, 106], BHR [107], alveolar nitric oxide concentrations [108], late phase sputum [109], and peripheral airway air trapping [110]. These improvements are associated with better asthma control [111–113], higher health-related quality of life [114–116] and better cost-effectiveness [116], along with reduced systemic exposure to inhaled corticosteroids [117], because comparable clinical effects can be obtained with a lower amount of delivered compound and with fewer unwanted effects .

Recently, the HFA-propelled extra-fine fixed combination formulation of beclomethasone dipropionate/formoterol (BDP/F) 100/6 μg has been developed [101, 118] and represents the only extrafine combination in both the pMDI and DPI formulations developed thus far [118].

Because of the small particle size of BDP/F, the two active drugs are delivered to both central and peripheral airways, resulting in a uniform treatment of inflammation and bronchoconstriction [119, 120]. In asthmatic patients BDP/F HFA significantly improved functional parameters reflecting small airway obstruction in comparison with equivalent non-extrafine inhaled formulations : forced vital capacity percent of predicted (%FVC, a simple indicator of small airways involvement) [121, 122], ventilation heterogeneity [123–125], peripheral airways resistance [126], local airway resistance obtained from computational fluid dynamics [127], BHR [124], and alveolar nitric oxide concentrations [127].

Huchon et al. showed that after 24 weeks of treatment extrafine BDP/F delivered by an HFA pMDI (400/24 μg) was superior in improving asthma control to the combination of the same drugs formulated as larger nonextrafine agents at equipotent doses (1000 μg BDP + 24 μg F) [128].

This is in line with results of several real-life studies [122, 129–132] showing that the use of extrafine-particle HFA-beclomethasone/formoterol was associated with a higher percentage of patients with well controlled asthma based on their Asthma Control Test and ACQ scores than the use of non-extrafine combination treatment. These improvements are associated with better health-related quality of life [122, 130, 131] and cost-effectiveness [132, 133]. Additionally, in most studies the Authors observed that the mean daily dose of iCSs was much higher for the large particle aerosols compared to the small particle aerosols of BDP/Form. Brusselle and Colleagues [134] have also shown that the benefits of small particle HFA-solution BDP/F aerosols on improving levels of disease control in non-smoking patients with asthma are also observed in asthmatic patients who currently smoke, reflecting real-life clinical practice, as often smoking asthmatic patients are excluded from randomized controlled clinical trials [134, 135].

Taken together, these studies show that extrafine-particle pressurized metered-dose inhalers might have additional clinical benefits in the treatment of asthma compared with coarse-particle treatment. Whether the clinical superiority in terms of control can be related to increased impact on distal lung abnormalities has not been demonstrated by an appropriately designed clinical study.

Montelukast is a systematically administered leukotriene receptor antagonist that reaches the small and large airways [136]. Leukotriene receptors are differently expressed in fibroblasts from peripheral compared to central airways [137], which may explain a suggested cysteinyl-leukotriene driven remodeling mainly in the peripheral airways and possibly resulting in a predominant effect of montelukast on the small airways. Several studies showed that biomarkers of peripheral airways are improved by montelukast: peripheral airways resistance [138, 139], air trapping [140–142], and alveolar nitric oxide [138, 144]. There is suggestive evidence that a improvement in distal dysfunction/inflammation after treatment with montelukast is associated with better asthma control and asthma-related Quality of Life in adults and children [138, 141–143].

Systemic parenteral treatment with omalizumab (an anti-immunoglobulin IgE monoclonal antibody) is used in selected patients with severe allergic asthma on treatment step 5 of asthma guidelines [9, 10]. In a prospective, time-series, single-arm observational study [145], 31 adult patients with severe refractory asthma despite the use of multiple controller medications, including high-dose iCSs (1432 ± 581 μg/d of fluticasone propionate equivalent), were enrolled. Alveolar nitric oxide (C_**alv**_NO) levels and airway-wall thickness as assessed by computed tomography significantly decreased at 48 weeks. Conversely, Pasha et al. did not find an association between the placebo and treatment groups in overall C_**alv**_NO levels or in the changes of C_**alv**_NO with time (may be for the initial low C_**alv**_NO levels in this asthmatic population) [146].

## Conclusions

Poor treatment compliance, failure to use inhalers correctly, heterogeneity of asthma phenotypes and associated comorbidities are the main contributing factors to poor disease control [20–24], but there is now a considerable amount of evidence supporting the concept that a higher level of small airway disease is associated with increased asthma symptoms, worse asthma control, more severe bronchial hyper-responsiveness, and an increased number of exacerbations [25]. Small-airways dysfunction is not only a feature of severe asthma : distal inflammation and remodeling are present in patients with all severities of disease [27–30]. Evidence is accumulating to support a distinct, “small aiways”, clinical phenotype for patients with uncontrolled asthma who have impaired small airway function and conventional measures of pulmonary function in the normal range [58]. There is also suggestive evidence that, in some “clinical” asthma phenotypes, the small airways are more affected, including nocturnal asthma, severe steroid-dependent or difficult-to-treat asthma, asthma complicated by smoking, elderly asthmatic patients and those with fixed airflow obstruction, and asthmatic children [147, 148]. Taken together, these findings support the view that distal lung is a very important target in any therapeutic strategy for effective treatment. The randomized clinical trials reported to date show that the extrafine and nonextrafine ICS formulations have similar efficacy in terms of primary endpoints; however the availability of small-particle aerosols enables a higher drug deposition into the peripheral lung [102–104] and potentially provides additional clinical benefits compared with large-particle treatment [147]. In several studies a better small-airways response to treatment with montelukast [142–144] or extrafine-particle ICSs [111–113] and ICS/LABA combination [128–132] is accompanied by better asthma control. Asthma is an inflammatory disease affecting the whole respiratory system, from central airways to lung parenchyma. Some patients have excellent control of disease with drugs that partially reach small airways, probably due to the heterogeneity of airway inflammation/dysfunction , but post hoc analysis of studies with non-extra-fine ICS/LABA combination to evaluate possible effects on small airways dysfunction are needed.

In addition to real-life studies, head to head (extrafine vs non-extrafine) randomized controlled studies are needed to evaluate whether changes in small-airway abnormalities correlate with improvement in clinical outcomes. Similarly, and even more importantly, clinical trials are needed to evaluate whether extrafine formulations would represent a specific therapeutic option for specific groups of patients (“clinical phenotypes”) characterized by enhanced small airway dysfunctions [148]. In summary, a proper small airways diagnostic assessment in routine clinical practice with early recognition of small-airways dysfunction is essential for an optimal management of asthma, particularly for early-stage diseases, when subjects are often asymptomatic and routine pulmonary function tests may be within normal ranges, and enables the physician to start treatment targeting the bronchial tree from beginning to end [149–151].
